# Circ_0000808 promotes the development of non-small cell lung cancer by regulating glutamine metabolism via the miR-1827/SLC1A5 axis

**DOI:** 10.1186/s12957-022-02777-x

**Published:** 2022-10-03

**Authors:** Yong Cai, Zhiyi Dong, Jiying Wang

**Affiliations:** 1 Department of Radiation Oncology, Shanghai Pulmonary Hospital, Tongji University School of Medicine, Shanghai, China; 2 Department of Traditional Chinese Medicine, Shanghai Pulmonary Hospital, Tongji University School of Medicine, Shanghai, China; 3 Department of Oncology, Shanghai Pulmonary Hospital, Tongji University School of Medicine, Shanghai, 200433 China

**Keywords:** Non-small cell lung cancer, circ_0000808, miR-1827, SLC1A5

## Abstract

**Background:**

Circular RNA (circRNA) has been proved to be an important molecular target for cancer treatment. However, the function and molecular mechanism of circ_0000808 in non-small cell lung cancer (NSCLC) are still unclear.

**Methods:**

Quantitative real-time PCR was used to detect the expression of circ_0000808, miR-1827, and solute carrier family 1 member 5 (SLC1A5). Cell proliferation, apoptosis, migration, and invasion were measured by cell counting kit 8 assay, colony formation assay, EdU staining, flow cytometry, wound healing assay, and transwell assay. The protein expression was measured by Western blot analysis. Dual-luciferase reporter assay and RIP assay were used to investigate the interactions between miR-1827 and circ_0000808 or SLC1A5. Cell glutamine metabolism was assessed by determining glutamine uptake, glutamate production, and α-ketoglutarate production. Xenograft mouse model was used to assess the in vivo effects of circ_0000808.

**Results:**

Circ_0000808 expression was upregulated in NSCLC tissues and cancer cells, and its silencing inhibited NSCLC cell proliferation, migration, and invasion and led to apoptosis. Further results confirmed that circ_0000808 interacted with miR-1827 to positively regulate *SLC1A5.* The rescue experiments showed that miR-1827 inhibitor reversed the suppressive effect of circ_0000808 knockdown on the malignant behaviors of NSCLC cells. Also, SLC1A5 overexpression abolished the inhibition effect of miR-1827 on NSCLC cell progression. In addition, circ_0000808/miR-1827/SLC1A5 axis positively regulated the glutamine metabolism process in NSCLC cells. Moreover, circ_0000808 knockdown reduced the NSCLC tumor growth in vivo.

**Conclusion:**

In summary, our data showed that circ_0000808 enhanced the progression of NSCLC by promoting glutamine metabolism through the miR-1827/SLC1A5 axis.

**Supplementary Information:**

The online version contains supplementary material available at 10.1186/s12957-022-02777-x.

## Introduction

Non-small cell lung cancer (NSCLC) is a common type of lung cancer [1]. At present, the treatment of NSCLC mainly adopts comprehensive treatment based on surgery, but it is a pity that most of the patients with NSCLC are diagnosed in the late stage, so they have lost the opportunity of surgery [2, 3]. Despite the continuous improvement of various medical treatment methods such as chemotherapy and radiotherapy, the prognosis of NSCLC patients is still unsatisfactory [4, 5]. Molecular targeted therapy drugs have the advantages of targeting, safety and convenience, and have achieved remarkable results in the treatment of cancer [6, 7]. Therefore, finding effective molecular targets is crucial for treating NSCLC.

Circular RNAs (circRNAs) are noncoding RNAs with circular structure formed by back-splicing [8, 9]. With the deepening of research, a variety of molecular mechanisms of circRNA have been discovered, of which the most studied is that it can regulate the microRNA (miRNA)/mRNA regulatory axis through acting as a miRNA sponge [10, 11]. Many studies have confirmed the important biological functions of circRNA, especially in human cancers [12]. Due to the stable properties of circRNA, circRNA has been found to be a potential biomarkers for the prognosis of many cancers [13, 14]. For example, circ_101237 was found to facilitate the proliferation and metastasis of NSCLC, suggesting that targeted inhibition of circ_101237 might be an effective measures to treat NSCLC [15]. Also, knockdown of circ_100676 was discovered to suppress NSCLC cell proliferation and metastasis [16]. Therefore, the study of circRNA may open up a new field for the molecular targeted therapy of NSCLC.

Circ_0000808 is a newly discovered circRNA and is derived from septin 9 (SEPT9) gene. In a recent study, Yu et al. used the circRNA microarray analysis and discovered that circ_0000808 was prominently upregulated in lung adenocarcinoma tissues [17]. In our previous pre-experiment, we confirmed that circ_0000808 was overexpressed in NSCLC tissues. However, the function of circ_0000808 in NSCLC has not been reported. Here, we explored the role of circ_0000808 in the proliferation, migration, invasion, and apoptosis of NSCLC through loss-functional test and revealed its possible molecular mechanism through further test verification.

## Materials and methods

### Samples collection

A total of 63 patients with NSCLC were recruited from the Shanghai Pulmonary Hospital, Tongji University School of Medicine, and their NSCLC tumor tissues and adjacent normal tissues were collected and stored at −80 °C. The clinicopathologic features in NSCLC patients were shown in Table 1. A part of fresh tissue was taken to prepare paraffin sections to perform immunohistochemical (IHC) staining using SP Kit (Invitrogen, Carlsbad, CA, USA) and anti-solute carrier family 1 member 5 (SLC1A5) (1:1600, Abcam, Cambridge, MA, USA). For this study, all patients signed written informed consent and received the approval from the Ethics Committee of the Shanghai Pulmonary Hospital, Tongji University School of Medicine.

**Table 1:** Correlation of the expression of circ_0000808 with clinicopathologic features in NSCLC patients

Parameters	*N* = 63	circ_0000808 expression		*p*-value
		High *N* = 31	Low *N* = 32	
Age, years				
≤ 60	25	12	13	0.719
> 60	38	20	18	
Sex				
Male	43	21	22	0.649
Female	20	11	9	
Smoking history				
Yes	39	20	19	0.921
No	24	12	12	
Tumor location				
Left lobe	27	15	12	0.513
Right lobe	36	17	19	
Tumor size				
≤ 3	30	10	20	0.008
> 3	33	22	11	
TNM stage				
I + II stage	49	21	28	0.018
III stage	14	11	3	
Lymph node metastasis				
No	43	15	28	< 0.001
Yes	20	17	3	

### Cell culture and transfection

Human NSCLC cell lines (HCC827, A549, and NCI-H1299) and normal bronchial epithelial cell line (BEAS-2B) were bought from ATCC (Manassas, VA, USA). PC9 cells were obtained from BioVector NTCC (Beijing, China). All NSCLC cells were cultured in RPMI-1640 medium (Gibco, Carlsbad, CA, USA), and BEAS-2B cells were grown in BEGM BulletKit (Lonza, Basel, Switzerland) at 37 °C with 5% CO_2_ incubator. All mediums contain 10% FBS (Gibco) and 1% penicillin (100 U/mL)-streptomycin (0.1 mg/mL) liquid (Gibco). The circ_0000808 lentivirus short hairpin RNA (sh-circ_0000808), miR-1827 mimic or inhibitor (miR-1827 or in-miR-1827), the pcDNA SLC1A5 overexpression vector, and their corresponding negative controls were constructed by RiboBio (Guangzhou, China). The above shRNA (50 nM), mimic (50 nM), inhibitor (50 nM), and vector (4.0 μg) were transfected into cells with Lipofectamine 3000 (Invitrogen) (0.75 μL per well in 24-well plates).

### Quantitative real-time PCR (qRT-PCR)

Total RNA was extracted by TRIzol Reagent (Invitrogen). After determining the RNA concentrations, cDNA Synthesis Kit (Invitrogen) was used to obtain cDNA. The cDNA was mixed with specific primers and SYBR Green (Solarbio, Beijing, China) to perform qRT-PCR. Relative expression was normalized by β-actin (for circ_0000808, SEPT9, and SLC1A5) or U6 (for miR-1827) and calculated by 2^−ΔΔCT^ method. Primer sequences were shown in Table 2.

**Table 2:** Primers sequences used for qRT-PCR

Name		Primers for PCR (5′-3′)
Circ_0000808	Forward	GCCTGAGCAAGGTGGTCAACATCAA
	Reverse	GGTGGCGGGGATGAAGTAGA
SEPT9	Forward	GGAGAGGGACCGGATCTCAG
	Reverse	CTTAGGGAGTCCACATGGCG
miR-1827	Forward	GTATGAGTGAGGCAGTAGAT
	Reverse	CTCAACTGGTGTCGTGGAG
SLC1A5	Forward	GAGACTCCAAGGGGCTCGC
	Reverse	CACAAGCAGGTTGGCTCGAAG
β-actin	Forward	TGGATCAGCAAGCAGGAGTA
	Reverse	TCGGCCACATTGTGAACTTT
U6	Forward	CTCGCTTCGGCAGCACA
	Reverse	AACGCTTCACGAATTTGCGT

### Identification of circRNA

In RNase R assay, the RNA extracted from A549 and NCI-H1299 cells was incubated with or without RNase R (Geneseed, Guangzhou, China) for 15 min, and then the RNA was used to perform qRT-PCR to measure circ_0000808 expression and linear RNA SEPT9 mRNA expression. In actinomycin D (ActD) assay, the cells were incubated with ActD solution (Seebio, Shanghai, China) for a certain period of time (0, 4, 8, 12, and 24 h). Then, the RNA was extracted, and qRT-PCR was carried out to examine circ_0000808 expression and linear RNA SEPT9 mRNA expression.

### Cell counting kit 8 (CCK8) assay

A549 and NCI-H1299 cells were seeded into 96-well plates (2 × 10^4^ cells/well) and cultured overnight. At the specified time point (0, 24, 48, and 72 h), the cells were added with CCK8 solution (Dojindo, Kumamoto, Japan) and further incubated for 4 h. The optical density (OD) value was measured at 450 nm to evaluate cell viability.

### Colony formation assay

A549 and NCI-H1299 cells were seeded in 6-well plates (200 cells/well). The cells were cultured at 37 °C for 14 days. After removing the cell medium, the colonies were fixed by 4% paraformaldehyde, stained by 0.1% crystal violet, and then counted under a microscope.

### EdU staining

According to the instructions of EdU Cell Proliferation Detection Assay Kit (Solarbio), EdU staining was performed to evaluate cell proliferation. Briefly, NSCLC cells were seeded in 96-well plates (1 × 10^4^ cells/well) and then incubated with EdU solution, Apollo staining reaction, and DAPI staining in turn. The fluorescents were visualized under a fluorescent microscope to count the EdU-positive cells (%).

### Flow cytometry

After cultured for 48 h, the transfected A549 and NCI-H1299 cells (2 × 10^5^ cells/well) were harvested and suspended with binding buffer. Then, cell suspensions were stained with Annexin V-FITC and PI (Vazyme, Nanjing, China) for 10 min in the dark. Under a FACScan flow cytometer, cell apoptotic rate (Annexin V+/PI− and Annexin V+/PI+) was assessed with CellQuest software. The excitation wavelength was 488 nm, and FITC fluorescence was detected by 515 nm wavelength, and PI was detected by a filter with a wavelength greater than 560 nm.

### Wound healing assay

After transfection, NSCLC cells were placed in 6-well plates (5 × 10^5^ cells/well). A wound was created on the cell layer with a 200 μL pipet tip after the cell fusion reached 90%. Then, the cells were replaced with serum-free medium. The cell wound was photographed under a microscope (40×, recorded as 0 h). After incubation for 24 h, the cell wound was photographed again under the microscope (40×). The percent wound closure rate was calculated to evaluate cell migration.

### Transwell assay

NSCLC cells (4 × 10^5^ cells/well) suspended with serum-free medium were seeded into the upper of Matrigel-coated transwell chambers (24-well; BD Biosciences, San Jose, CA, USA), and the serum medium was added into the lower chambers. After 24 h, the number of invaded cells was counted under a microscope (100×).

### Western blot (WB) analysis

The cells were lysed by RIPA buffer (Beyotime, Shanghai, China). After determining the protein concentration, the protein was separated by SDS-PAGE gel followed by transferred to PVDF membranes. After hatched with skim milk, the membrane was treated with primary antibodies and secondary antibody (ab205718, 1:50,000, Abcam) in turn. The protein signals were visualized by BeyoECL Plus kit (Beyotime), and relative protein expression was analyzed via Image Lab software. Primary antibodies include anti-ki67 (1:1000, Abcam) [18], anti-Bax (1:1000, Abcam) [18], anti-E-cadherin (1:100, Abcam) [19], anti-SLC1A5 (1:1000, Abcam) [20], and anti-β-actin (1:2000, Abcam) [18].

### Dual-luciferase reporter assay

According to the binding sites between miR-1827 and circ_0000808 or SLC1A5 3′UTR predicted by the bioinformatics software, the wild-type (WT) and mutant-type (MUT) circ_0000808 and SLC1A5 3′UTR vectors were constructed through sub-cloning their sequences into the psiCHECK2 reporter vector. The vectors were transfected into A549 and NCI-H1299 cells with miR-1827 mimic or miR-NC. After 48 h, Dual-Luciferase Reporter Assay System (Solarbio) was used to detect relative luciferase activity.

### RIP assay

The cell lysates were incubated with RNA magnetic beads (Millipore, Billerica, MA, USA) conjugated with anti-Ago2 (1:200, Abcam) or anti-IgG (1:200, Solarbio). Then, the immunoprecipitated RNA was subjected to qRT-PCR to analyze the circ_0000808 and miR-1827 expression.

### Measurement of cell glutamine metabolism

Glutamine uptake, glutamate production, and α-ketoglutarate production were analyzed to evaluate cell glutamine metabolism according to the protocols of Glutamine Assay Kit (Abcam), Glutamate Assay Kit (Abcam), and α-ketoglutarate Assay Kit (Abcam), respectively.

### Mice xenograft models

Male 5-week-old BALB/c mice (Vital River, Beijing, China) were randomly divided into 2 groups (*n* = 5). NCI-H1299 cells were transfected with sh-circ_0000808 or sh-NC. Then, the viral supernatant was used for infecting NCI-H1299 cells. After 48 h, infected cells were selected using puromycin (2 mg/mL) for 3 days. NCI-H1299 cells stable transfected with sh-circ_0000808 or sh-NC were subcutaneously injected into the flank of mice. Tumor volume were calculated every 7 days using the formula length × width^2^/2, and tumor tissues were harvested at 35 days. The tumor tissues were used to determine the circ_0000808, miR-1827, and SLC1A5 expression using qRT-PCR and to measure SLC1A5 protein expression using WB analysis. In addition, paraffin sections of tumor tissues were prepared, and then, IHC staining was performed using SP Kit (Invitrogen) and specific antibodies, including anti-ki67 (1:200, Abcam), anti-Bax (1:50, Abcam), anti-E-cadherin (1:500, Abcam), and anti-SLC1A5 (1:1600, Abcam). Animal experiments were approved by the Animal Ethics Committee of the Shanghai Pulmonary Hospital, Tongji University School of Medicine.

### Statistical analysis

Data were presented as mean ± SD from at least three independent experiments. Student’s *t*-test or analysis of variance was performed for group comparisons. Pearson correlation analysis was utilized for assessing the correlations among circ_0000808, miR-1827, and SLC1A5. GraphPad Prism 6.0 software was used for data analysis. *P* < 0.05 was considered as significant.

## Results

### Circ_0000808 was overexpressed in NSCLC tissues and cells

**Fig. 1:**
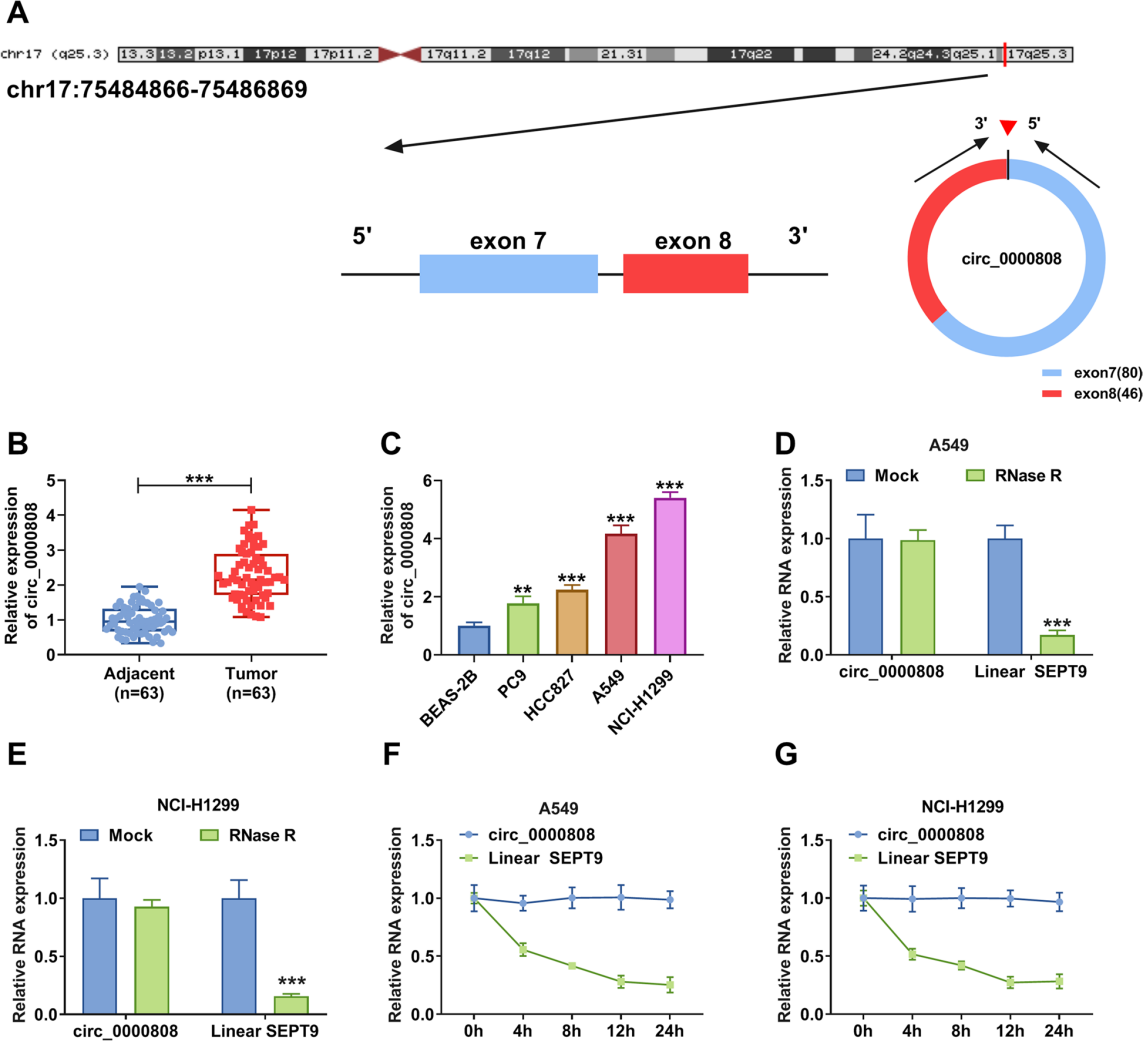
Circ_0000808 was overexpressed in NSCLC tissues and cells. **A** The basic information of circ_0000808 was shown. **B** The expression of circ_0000808 in NSCLC tumor tissues and adjacent normal tissues was measured by qRT-PCR. **C** The expression of circ_0000808 was examined by qRT-PCR in tumor tissues of NSCLC patients with different TNM stages. **D** QRT-PCR was used to measure circ_0000808 expression in tumor tissues of patients with or without lymph node metastasis. **E** The circ_0000808 expression in NSCLC cells (PC9, HCC827, A549, and NCI-H1299) and BEAS-2B cells was detected by qRT-PCR. RNase R assay (**F**) and ActD assay (**G**) were performed to assess the circular characteristic of circ_0000808. **P < 0.01, ***P < 0.001

Circ_0000808 is located at chr17:7548466-75486869 and is formed by the back-splicing of exons 7–8 of SEPT9 gene (Fig. 1A). Through detecting circ_0000808 expression in NSCLC tumor tissues (*n* = 63) and adjacent normal tissues (*n* = 63), we discovered that circ_0000808 was significantly upregulated in NSCLC tumor tissues (Fig. 1B). Through analyzing, we confirmed that circ_0000808 expression was associated with the tumor size, TNM stage, and lymph node metastasis in patients with NSCLC (Table 1). Circ_0000808 expression was higher in tumor tissues of patients with stage 3 than that in patients with stages 1–2 (Fig. 1C), and it was higher in tumor tissues of patients with lymph node metastasis than that in patients without lymph node metastasis (Fig. 1D). In four NSCLC cells, circ_0000808 expression also was higher than that in normal bronchial epithelial cell line (BEAS-2B) (Fig. 1E). Further experiments showed that circ_0000808 could resist the digestion of RNase R, while its linear RNA SEPT9 could be digested by RNase R (Fig. 1F). After ActD treatment, we observed that the stability of circ_0000808 was higher than that of linear RNA SEPT9 (Fig. 1G). These data confirmed that circ_0000808 had a circular structure, which might play an important role in NSCLC.

### Silencing of circ_0000808 suppressed NSCLC cell proliferation, migration, and invasion

**Fig. 2:**
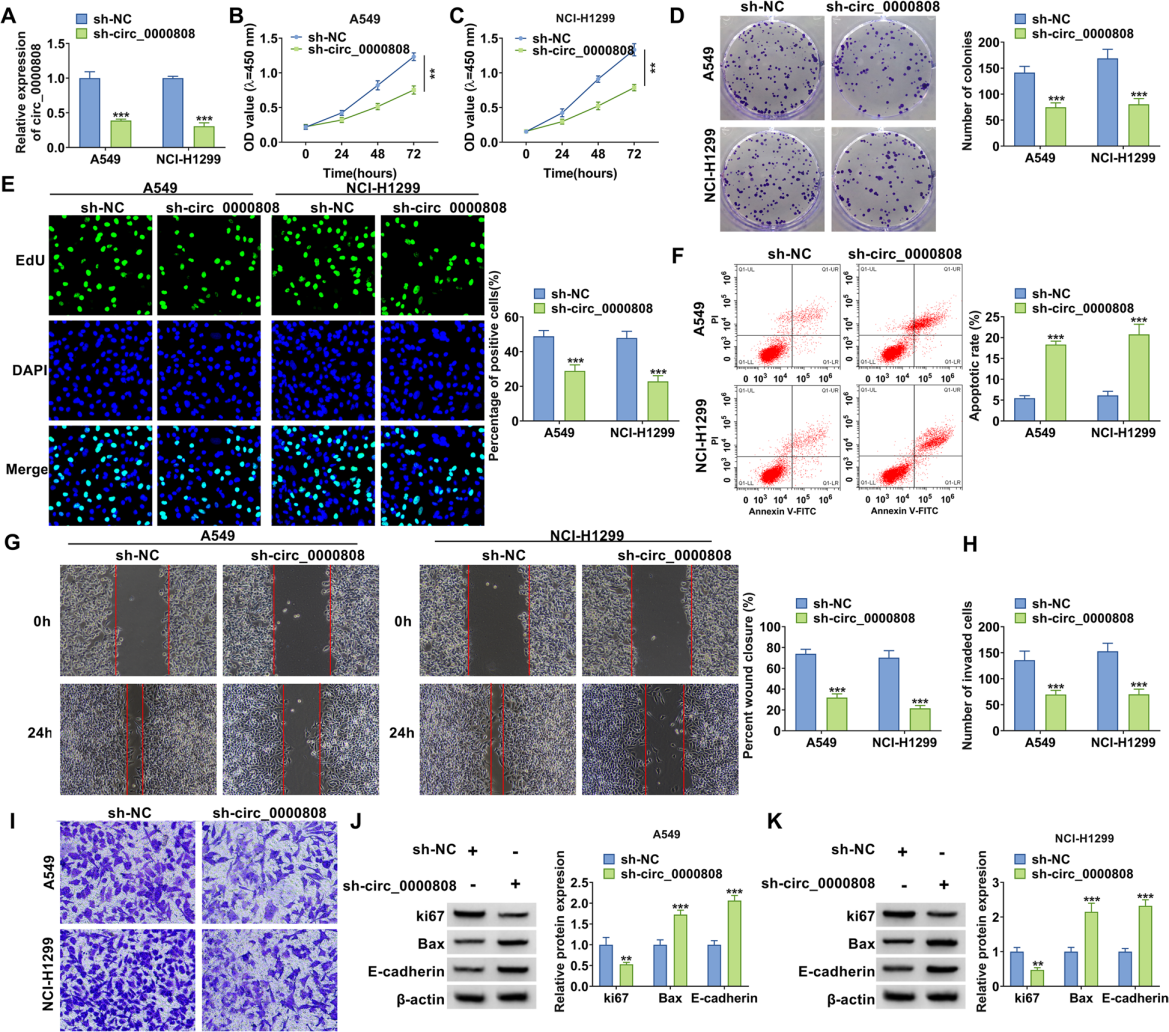
Effects of sh-circ_0000808 on NSCLC cell proliferation, apoptosis, migration, and invasion. A549 and NCI-H1299 cells were transfected with sh-NC or sh-circ_0000808. **A** The expression of circ_0000808 was measured by qRT-PCR. CCK8 assay (**B**–**C**), colony formation assay (**D**), and EdU staining (**E**) were performed to assess cell proliferation. Flow cytometry (**F**), wound healing assay (**G**), and transwell assay (H-I) were used to examine cell apoptosis, migration, and invasion (**J**–**K**). The protein levels of ki67, Bax, and E-cadherin were determined by WB analysis. **P < 0.01, ***P < 0.001

To confirm the role of circ_0000808 in NSCLC, sh-circ_0000808 was used to reduce circ_0000808 expression in NSCLC cells (A549 and NCI-H1299). After transfected with sh-circ_0000808 into A549 and NCI-H1299 cells, circ_0000808 expression was indeed markedly decreased (Fig. 2A). CCK8 assay, colony formation assay, and EdU staining were used to measure NSCLC cell proliferation, and the results showed that circ_0000808 knockdown reduced the viability, the colony numbers, and the EdU-positive cells (Fig. 2 B–E). The detection results of apoptosis rate confirmed that circ_0000808 silencing could promote the apoptosis of A549 and NCI-H1299 cells (Fig. 2F). Besides, knockdown of circ_0000808 inhibited the wound closure rate and the invaded cell numbers in A549 and NCI-H1299 cells (Fig. 2 G–I). WB analysis was used to detect marker protein expression, and the results indicated that circ_0000808 silencing decreased the protein expression of proliferation marker ki67 while promoted the protein expression of apoptosis marker Bax and metastasis marker E-cadherin in A549 and NCI-H1299 cells (Fig. 2 J–K). In addition, we confirmed that circ_0000808 silencing also repressed the expression of ZEB1 and vimentin in NSCLC cells (Supplementary Fig. 1A). These data illuminated that circ_0000808 might facilitate NSCLC progression.

### Circ_0000808 acted as miR-1827 sponge

**Fig. 3:**
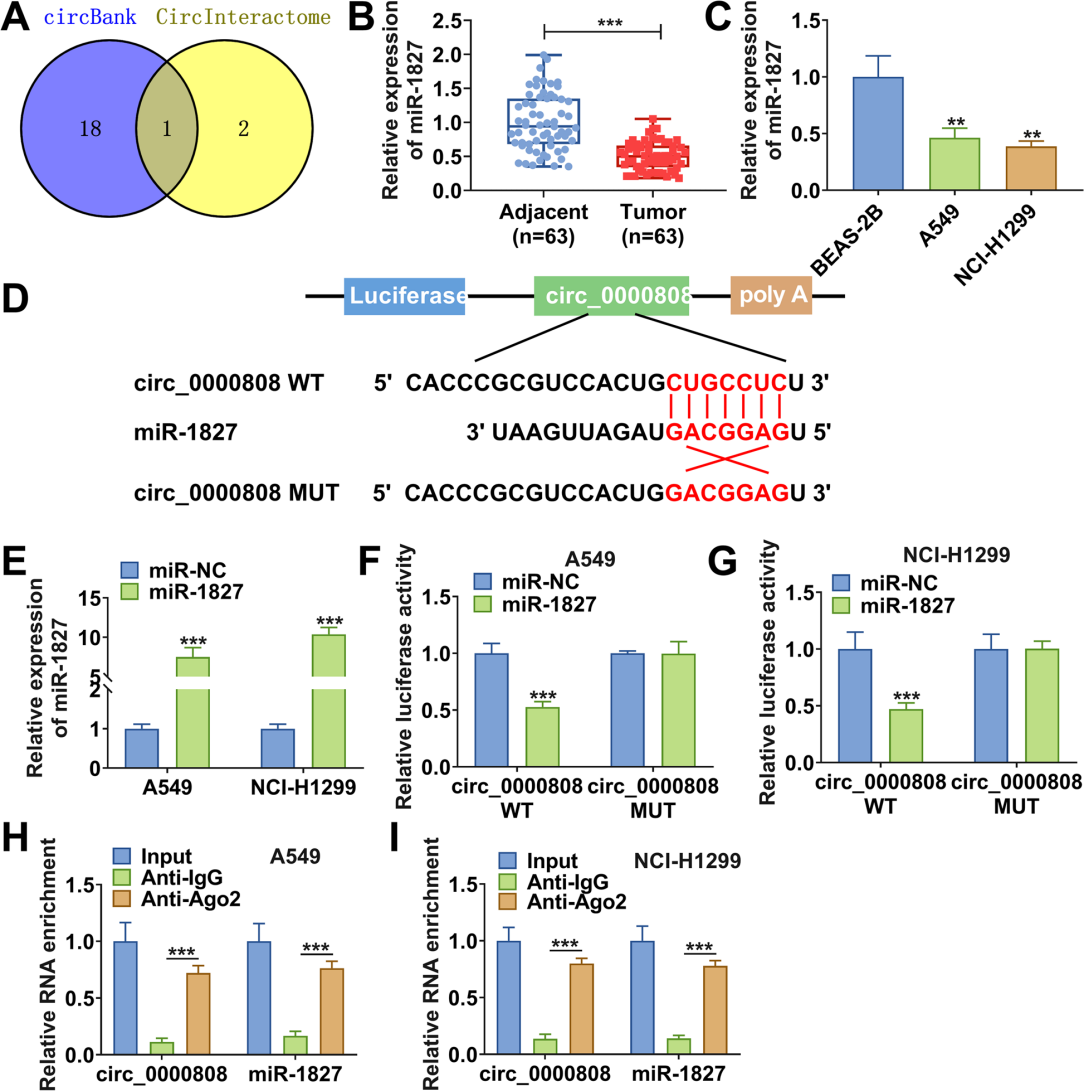
Circ_0000808 acted as miR-1827 sponge. **A** Venn diagram showed the targeted miRNAs of circ_0000808 predicted by both CircInteractome software and circBank software. **B** The expression of miR-1827 in NSCLC tumor tissues and adjacent normal tissues was measured by qRT-PCR. **C** The miR-1827 expression in NSCLC cells (A549 and NCI-H1299) and BEAS-2B cells was detected by qRT-PCR. **D** The sequences of circ_0000808 WT/MUT vectors were shown. **E** The transfection efficiency of miR-1827 mimic was confirmed by detecting miR-1827 expression using qRT-PCR. Dual-luciferase reporter assay (F-G) and RIP assay (H-I) were used to assess the interaction between circ_0000808 and miR-1827. **P < 0.01, ***P < 0.001

Using the CircInteractome software and circBank software, we predicted the targeted miRNA of circ_0000808 and found that miR-1827 possessed complementary binding sites with circ_0000808 (Fig. 3A). In NSCLC tissues and cells, a significant lowly expressed miR-1827 was observed compared to adjacent normal tissues and BEAS-2B cells (Fig. 3 B–C). According to the binding sites between circ_0000808 and miR-1827, the circ_0000808 WT/MUT vectors were constructed (Fig. 3D). After confirming that miR-1827 mimic indeed promoted miR-1827 expression (Fig. 3E), miR-1827 mimic and the circ_0000808 WT/MUT vectors were co-transfected into A549 and NCI-H1299 cells. Dual-luciferase reporter assay results verified that miR-1827 overexpression only reduced the luciferase activity of circ_0000808 WT vector without affecting that of the MUT vector (Fig. 3 F–G). Moreover, RIP assay results revealed that circ_0000808 and miR-1827 could be markedly enriched in anti-Ago2 (Fig. 3 H–I). These data determined that circ_0000808 could interact with miR-1827.

### The regulation of circ_0000808 silencing on NSCLC proliferation, apoptosis, migration, and invasion could be reversed by miR-1827 inhibitor

**Fig. 4:**
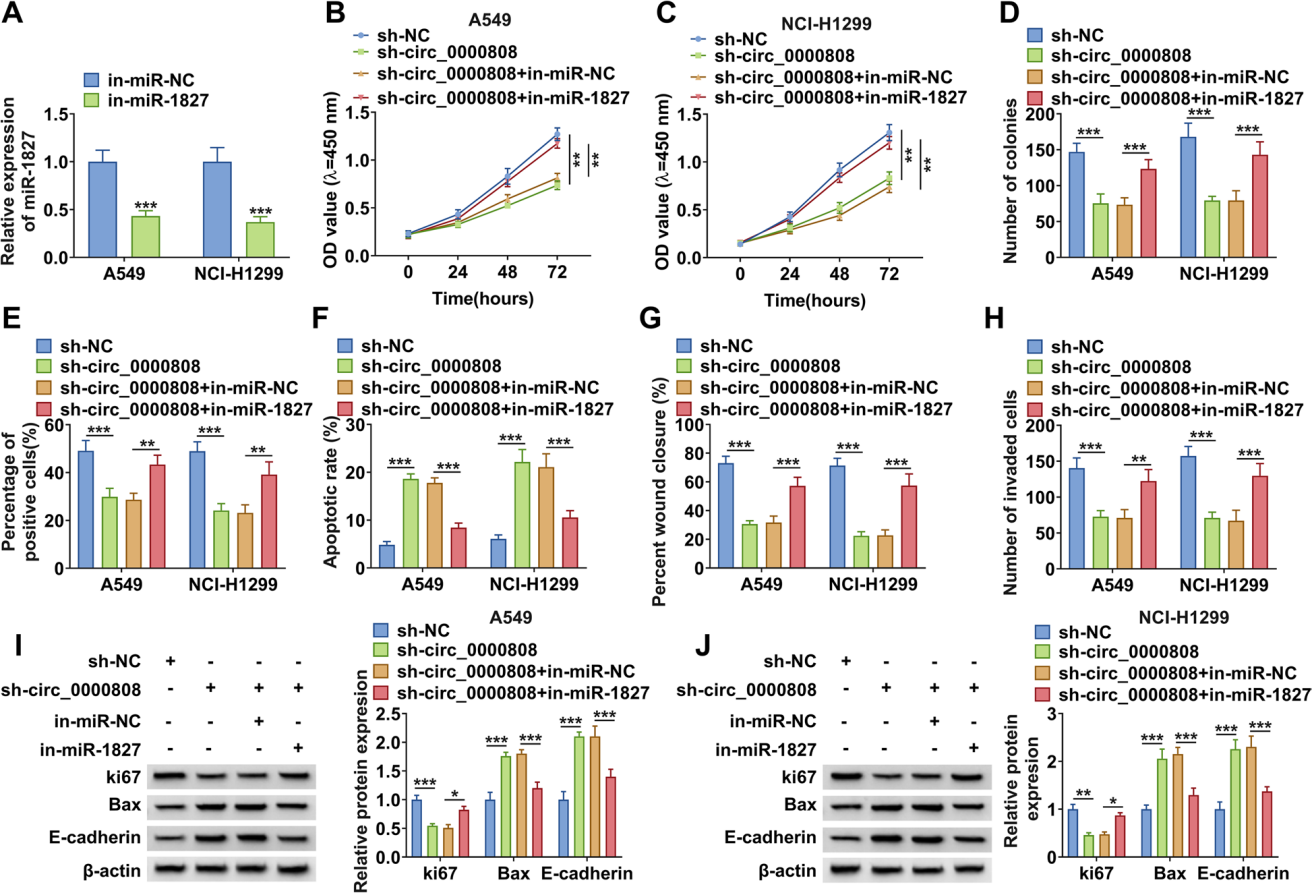
Effects of sh-circ_0000808 and in-miR-1827 on NSCLC cell proliferation, apoptosis, migration, and invasion. **A** The transfection efficiency of in-miR-1827 was confirmed by measuring miR-1827 expression using qRT-PCR. **B**–**J** A549 and NCI-H1299 cells were transfected with sh-NC, sh-circ_0000808, sh-circ_0000808 + in-miR-NC, or sh-circ_0000808 + in-miR-1827. Cell proliferation was determined using CCK8 assay (**B**–**C**), colony formation assay (**D**), and EdU staining (**E**). Cell apoptosis, migration, and invasion were evaluated using flow cytometry (**F**), wound healing assay (**G**), and transwell assay (**H**). **I**–**J** WB analysis was used to examine the protein levels of ki67, Bax, and E-cadherin. *P < 0.05, **P < 0.01, ***P < 0.001

The rescue experiments were performed to explore whether circ_0000808 regulated NSCLC progression by sponging miR-1827. The in-miR-1827 was constructed and transfected into A549 and NCI-H1299 cells. The decreased miR-1827 expression confirmed the transfection efficiency of in-miR-1827 (Fig. 4A). Then, sh-circ_0000808 and in-miR-1827 were co-transfected into A549 and NCI-H1299 cells. The inhibitory effects of circ_0000808 knockdown on the viability, the colony number, and the EdU positive cells could be abolished by in-miR-1827 (Fig. 4 B–E and Supplementary Fig. 2 A–B). Also, inhibition of miR-1827 also reversed the promotion effect of circ_0000808 knockdown on the apoptosis of NSCLC cells (Fig. 4F and Supplementary Fig. 2C). Furthermore, the negative regulation of circ_0000808 silencing on the migration and invasion of A549 and NCI-H1299 cells also could be overturned by miR-1827 inhibitor (Fig. 4 G–H and Supplementary Fig. 2 D–E). The detection of protein expression confirmed that the decrease effect of circ_0000808 knockdown on ki67 protein level and the increase effect on Bax and E-cadherin protein levels could be reversed by the addition of in-miR-1827 (Fig. 4 I–J). All results indicated that circ_0000808 sponged miR-1827 to mediate NSCLC progression.

### MiR-1827 directly targeted SLC1A5

**Fig. 5:**
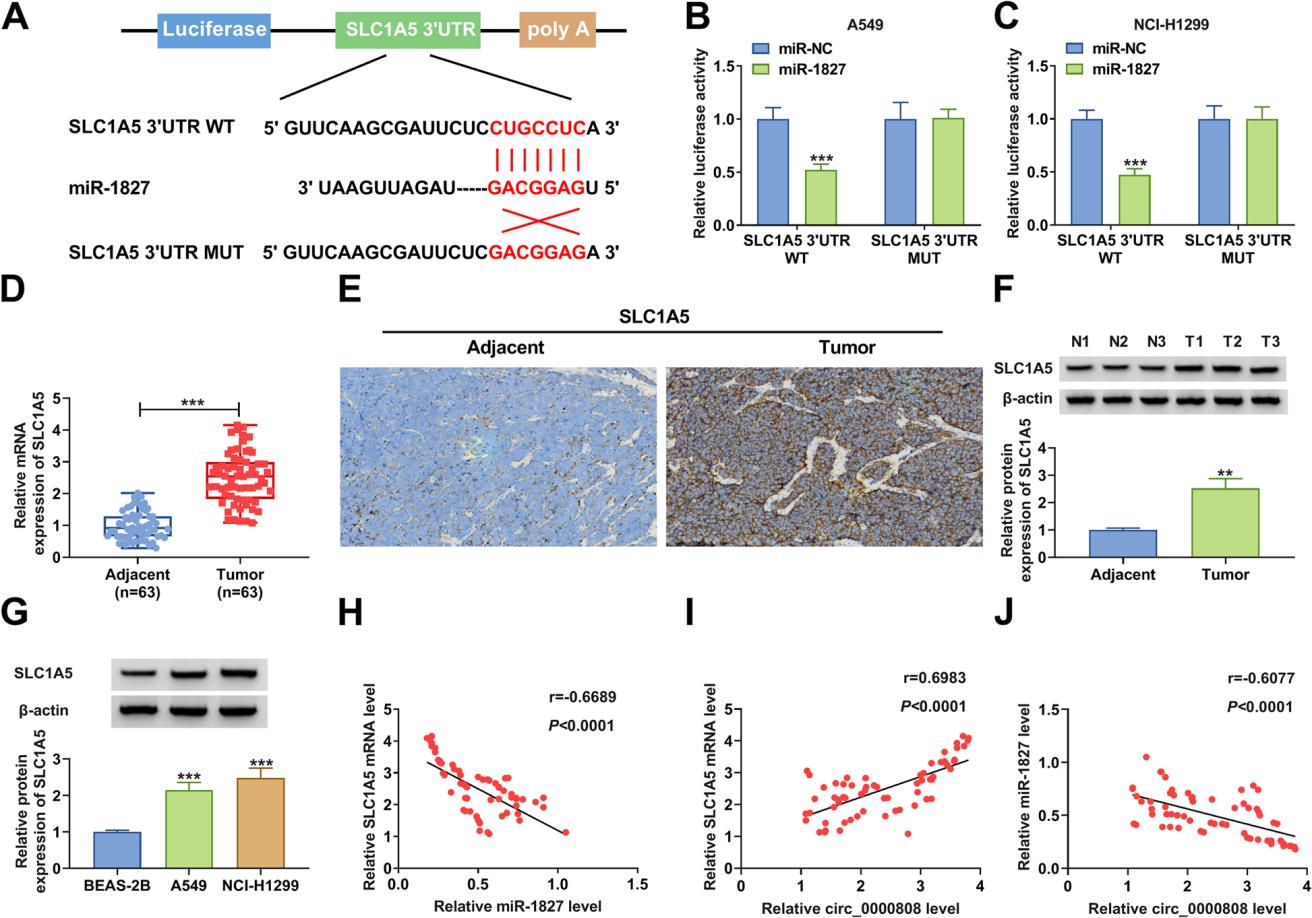
MiR-1827 directly targeted SLC1A5. **A** The sequences of SLC1A5 3′UTR WT/MUT vectors were shown. **B**–**C** Dual-luciferase reporter assay was used to verify the interaction between SLC1A5 and miR-1827. **D** The SLC1A5 mRNA expression in NSCLC tumor tissues and adjacent normal tissues was measured by qRT-PCR. **E** IHC staining was used to determine the SLC1A5-positive cells in NSCLC tumor tissues and adjacent normal tissues. **F** The SLC1A5 protein expression in NSCLC tumor tissues and adjacent normal tissues was analyzed using WB analysis. **G** WB analysis was performed to test the SLC1A5 protein expression in NSCLC cells (A549 and NCI-H1299) and BEAS-2B cells. **H**–**J** The correlations among circ_0000808, miR-1827, and SLC1A5 expression in NSCLC tumor tissues were analyzed by Pearson correlation analysis. **P < 0.01, ***P < 0.001

The target of miR-1827 was predicted using the TargetScan software to perfect the hypothesis of the circRNA/miRNA/mRNA axis. The 3′UTR of SLC1A5 was found to have binding sites with miR-1827. Then, the SLC1A5 3′UTR WT/MUT vectors were constructed (Fig. 5A). After transfected with the miR-1827 mimic and the reporter vectors into A549 and NCI-H1299 cells, we discovered that miR-1827 mimic reduced the luciferase activity of SLC1A5 3′UTR WT vector, while had no effect on that of the MUT vector (Fig. 5 B–C). These data confirmed the interaction between miR-1827 and SLC1A5. By detecting SLC1A5 mRNA expression, we confirmed that SLC1A5 expression was upregulated in NSCLC tumor tissues (Fig. 5D). IHC staining results showed that the SLC1A5-positive cells were increased in NSCLC tumor tissues compared to adjacent normal tissues (Fig. 5E). In NSCLC tumor tissues and cells, we also found the significantly overexpressed SLC1A5 at the protein level (Fig. 5 F–G). Additionally, correlation analysis showed that SCL1A5 mRNA expression was negatively correlated with miR-1827 expression and positively correlated with circ_0000808 expression in NSCLC tumor tissues (Fig. 5 H–I). Also, there had a negatively correlation between miR-1827 and circ_0000808 (Fig. 5J). Therefore, we confirmed that circ_0000808 could sponge miR-1827 to positively regulate SCL1A5.

### MiR-1827 inhibited NSCLC progression by targeting SLC1A5

**Fig. 6:**
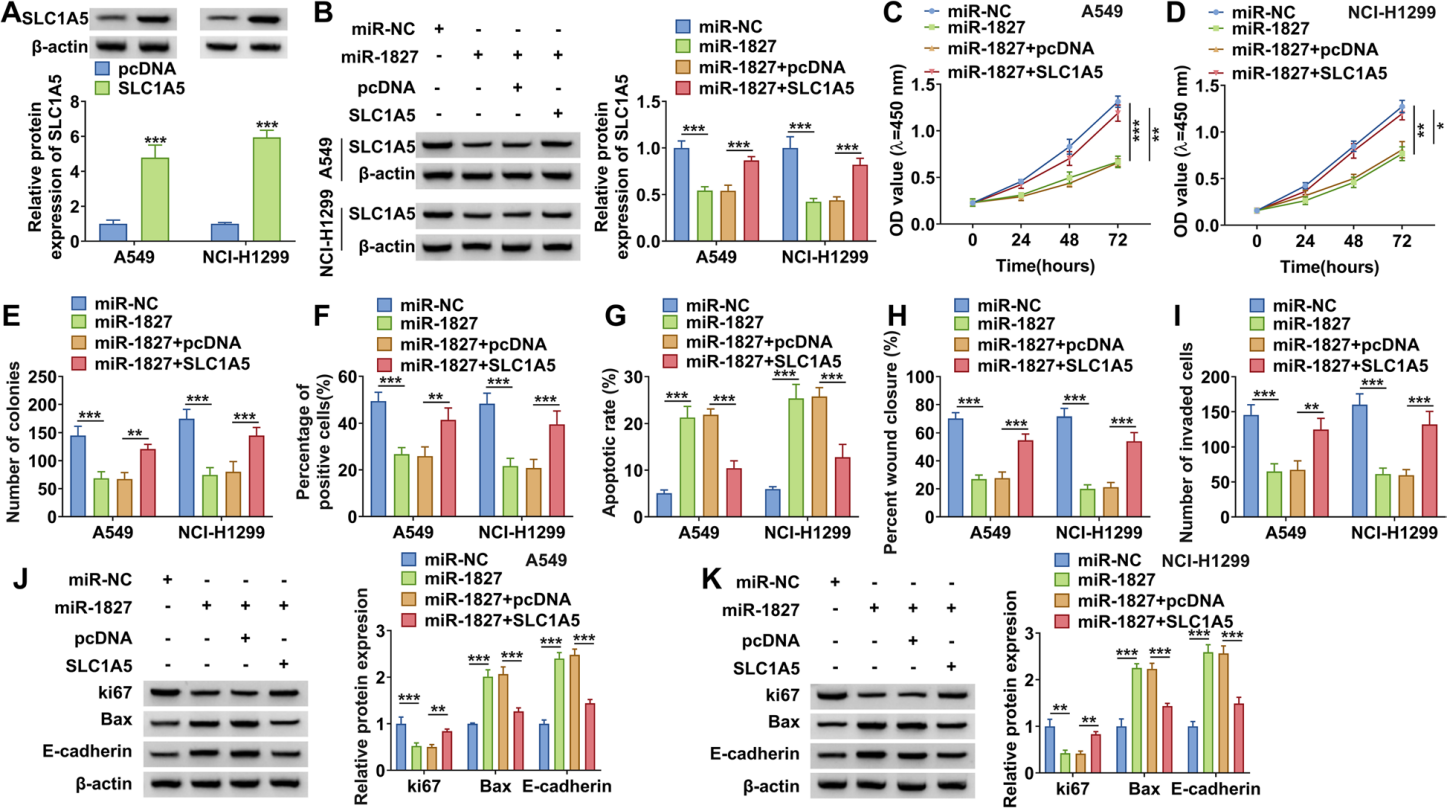
Effects of miR-1827 and SLC1A5 on NSCLC cell proliferation, apoptosis, migration, and invasion. **A** The transfection efficiency of pcDNA SLC1A5 overexpression vector was confirmed by measuring SLC1A5 protein expression by WB analysis. **B**–**K**) A549 and NCI-H1299 cells were transfected with miR-NC, miR-1827, miR-1827 + pcDNA, or miR-1827 + SLC1A5. **B** The protein expression of SLC1A5 was measured by WB analysis. CCK8 assay (**C**–**D**), colony formation assay (**E**), and EdU staining (**F**) were performed to detect cell proliferation. Cell apoptosis, migration, and invasion were determined by flow cytometry (**G**), wound healing assay (**H**), and transwell assay (**I**). **J**–**K** The protein levels of ki67, Bax, and E-cadherin were measured by WB analysis. *P < 0.05, **P < 0.01, ***P < 0.001

After confirmed the transfection efficiency of pcDNA SLC1A5 overexpression vector (Fig. 6A), we co-transfected with miR-1827 mimic and pcDNA SLC1A5 overexpression vector into A549 and NCI-H1299 cells to perform rescue experiments. As shown in Fig. 6B, the decreasing effect of miR-1827 mimic on SLC1A5 protein expression could be abolished by pcDNA SLC1A5 overexpression vector. Through assessing cell viability, colony numbers, and the EdU-positive cells, we found that miR-1827 overexpression could repress NSCLC cell proliferation, while this effect could be reversed by SLC1A5 overexpression (Fig. 6 C–F and Supplementary Fig. 3 A–B). Besides, the promotion effect of miR-1827 on cell apoptosis rate and the suppressive effect on the wound closure rate and the invaded cell number also could be overturned by overexpressing SLC1A5 (Fig. 6 G–I and Supplementary Fig. 3 C–E). In addition, miR-1827 overexpression reduced ki67 protein expression while increased the Bax and E-cadherin protein expression. However, these effects also could be reversed by SLC1A5 overexpression (Fig. 6 J–K). Hence, we confirmed that miR-1827 targeted SLC1A5 to regulate NSCLC proliferation, apoptosis, migration, and invasion.

### Circ_0000808/miR-1827/SLC1A5 axis mediated glutamine metabolism

**Fig. 7:**
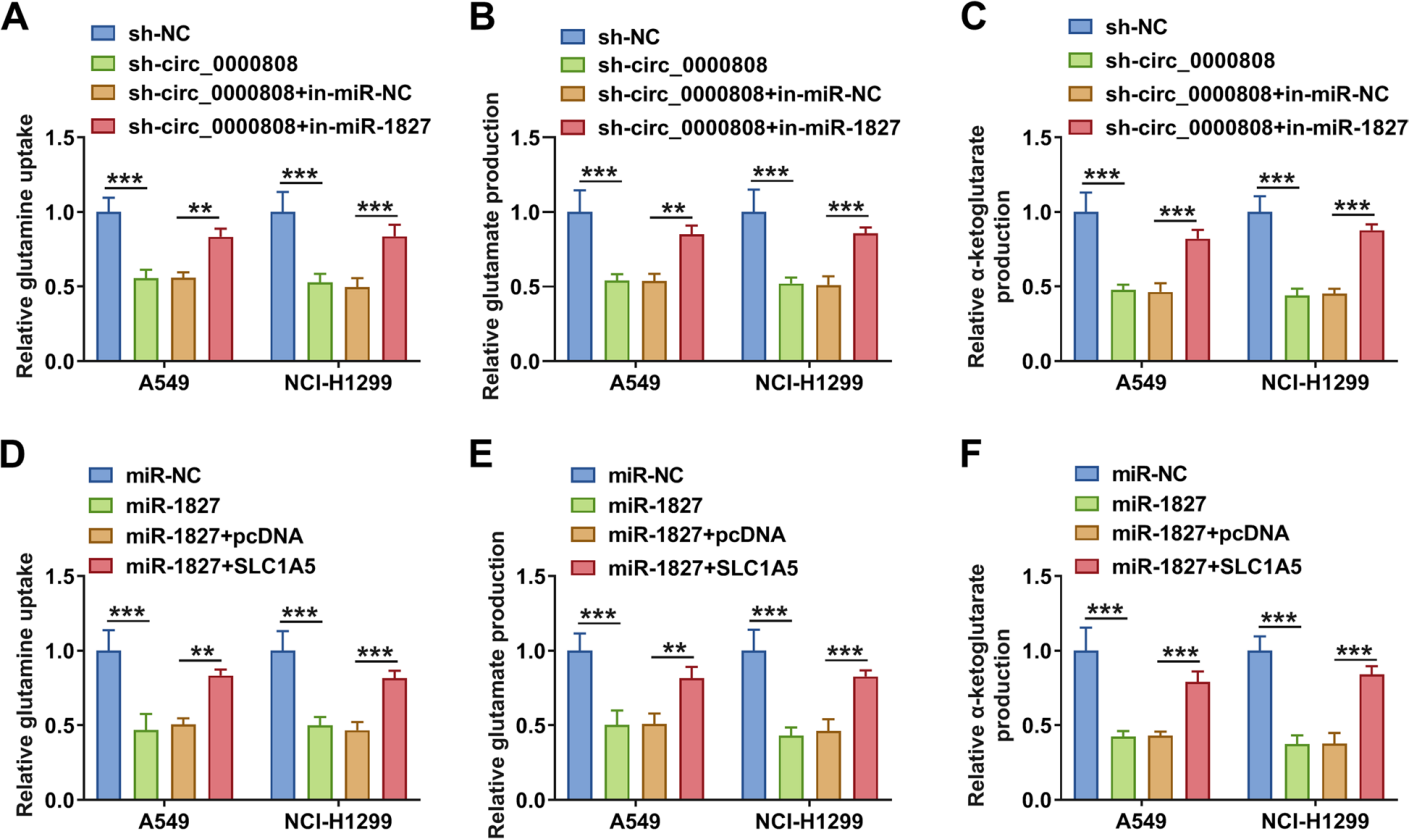
Circ_0000808/miR-1827/SLC1A5 axis mediated glutamine metabolism. Under different transfection conditions, glutamine uptake (**A** and **D**), glutamate production (**B** and **E**), and α-ketoglutarate production (**C** and **F**) were determined by corresponding assay kits. **P < 0.01, ***P < 0.001

As a glutamine transporter, SLC1A5 activity has been shown to be related to the intensity of intracellular glutamine metabolism. Here, we assessed the glutamine metabolism of NSCLC cells. Our data showed that circ_0000808 knockdown could inhibit the glutamine uptake, glutamate production, and α-ketoglutarate production of A549 and NCI-H1299 cells, while miR-1827 inhibitor could reverse these results (Fig. 7 A–C). Moreover, miR-1827 overexpression also had an inhibition effect on the glutamine uptake, glutamate production, and α-ketoglutarate production of A549 and NCI-H1299 cells, and this effect could be abolished by overexpressing SLC1A5 (Fig. 7 D–F). Therefore, we hypothesized that circ_0000808 might promote the glutamine metabolism process by mediating the miR-1827/SLC1A5 axis and thus facilitating the progression of NSCLC.

### Downregulated circ_0000808 reduced NSCLC tumorigenesis

**Fig. 8:**
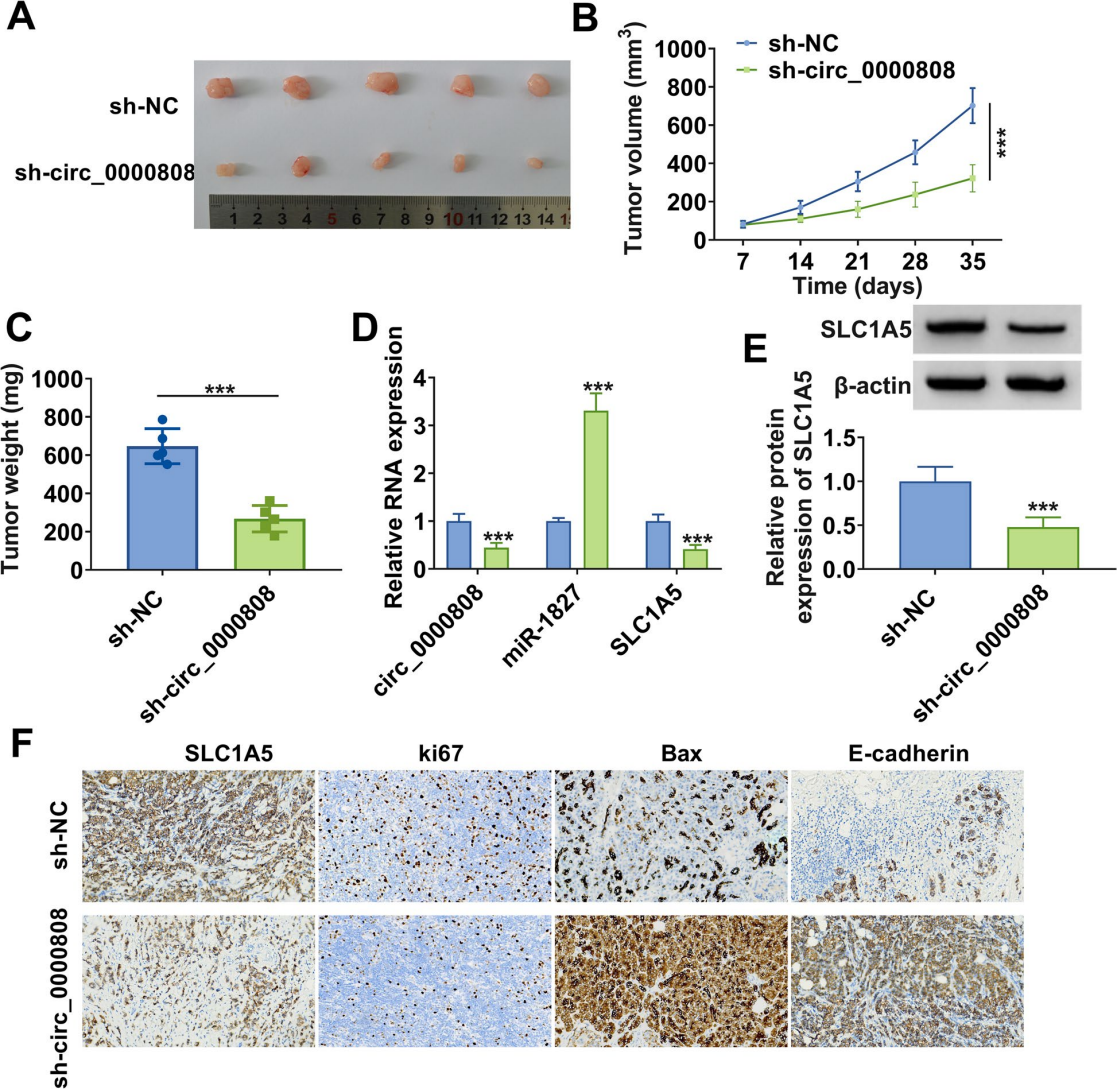
Downregulated circ_0000808 reduced NSCLC tumorigenesis. NCI-H1299 cells transfected with sh-NC or sh-circ_0000808 were injected into nude mice. **A** Images of tumors in each group. **B** Tumor volume was measured every 7 days. **C** Tumor weight was detected after 35 days. **D** The expression of circ_0000808, miR-1827, and SLC1A5 in the tumor tissues of each group was determined by qRT-PCR. **E** The protein expression of SLC1A5 in the tumor tissues of each group was examined by WB analysis. **F** IHC staining was used to assess the positive cells of SLC1A5, ki67, Bax, and E-cadherin in the tumor tissues of each group. ***P < 0.001

In addition, the xenograft tumor was constructed to further confirm the role of circ_0000808 in NSCLC tumorigenesis in vivo. Figure 8A showed the tumor images of each group, and it could be seen that the tumor size of the sh-circ_0000808 group was significantly smaller than that of the control group. By measuring tumor volume and weight, we determined that tumor volume and weight in the sh-circ_0000808 group were remarkably lower than those in the control group (Fig. 8 B–C). In the tumor tissues of sh-circ_0000808 group, we found that circ_0000808 and SLC1A5 expression was enhanced, while miR-1827 expression was decreased (Fig. 8D). Also, a significant reduced SLC1A5 protein expression was discovered in the tumor tissues of sh-circ_0000808 group (Fig. 8E). Moreover, IHC staining was performed on the tumor tissues of each group, and the results showed that the positive cells of SLC1A5 and ki67 were decreased, while the positive cells of Bax and E-cadherin were increased in the tumor tissues of sh-circ_0000808 group (Fig. 8F). Besides, circ_0000808 silencing also had an inhibition on the expression of ZEB1 and vimentin in tumor tissues (Supplementary Fig. 1B). All data revealed that circ_0000808 played a pro-tumor role in NSCLC.

## Discussion

At present, there is no clear clinical result on the etiology of NSCLC. A widely accepted view is that a variety of risk factors lead to a series of genetic changes, which then trigger normal cells to become cancerous [21, 22]. In NSCLC, many circRNAs have been proved to act as pro-cancer factors and anticancer factors to mediate the regulation of malignant progression of cancer, such as circP4HB [23] and circ_100146 [24] as oncogenes while circPTPRA [25] and circ_0002483 as tumor suppressor [26]. Circ_0000808 is a novel circRNA that has not been reported yet. Here, the role of circ_0000808 in NSCLC was studied for the first time. Our study indicated that circ_0000808 was overexpressed in NSCLC tissues and cells, which in line with the results of previous microarray analysis [17]. Importantly, we confirmed that circ_0000808 had a stable circular structure, which provided a necessary condition for it to be a therapeutic target for NSCLC. Loss-of-function experiments showed that circ_0000808 knockdown suppressed NSCLC cells proliferation, migration, and invasion while promoted apoptosis in vitro. Also, animal experiments indicated that downregulated circ_0000808 restrained NSCLC tumorigenesis in vivo. The above information enlightened us that circ_0000808 might be utilized as a potential therapeutic target for NSCLC.

The idea that circRNAs can act as miRNA sponges has been widely confirmed in many past studies. In this, we confirmed that circ_0000808 could act as miR-1827 sponge in NSCLC. MiR-1827 acted as a tumor suppressor to hinder the development of many cancers, including hepatocellular carcinoma [27, 28], colorectal cancer [29], and endometrial cancer [30]. In the past research, miR-1827 had been found to inhibit the lung cancer cell growth, metastasis, and angiogenesis [31–33]. Consistent with the previous results, our study also verified the anti-tumor role of miR-1827 in NSCLC, which was manifested in decreased cell proliferation, migration, and invasion ability while increased apoptosis. In the rescue experiments, our data uncovered that the negative regulation of sh-circ_0000808 on NSCLC progression was abolished by in-miR-1827, confirming the conclusion that circ_0000808 targeted miR-1827 to mediate NSCLC progression.

Glutamine metabolism is one of the important sources of energy for cancer cells [34]. High levels of glutamine in the blood provide a ready source of carbon and nitrogen to support cancer cell biosynthesis, energy metabolism, and homeostasis, thereby promoting tumor growth [35, 36]. SLC1A5 is an important transporter of glutamine, which mainly transports glutamine into cells [37]. In a variety of cancers, targeted inhibition of SLC1A5 has been shown to reduce glutamine metabolism and thereby inhibiting cancer progression [38]. More importantly, SLC1A5-mediated glutamine metabolism has been confirmed to be critical to the development of NSCLC [39, 40]. In this, we proposed that SLC1A5 could be targeted by miR-1827, and circ_0000808 positive regulated SLC1A5 by sponging miR-1827. Our data revealed that miR-1827 suppressed NSCLC proliferation, migration, invasion, and enhanced apoptosis through targeting SLC1A5. By assessing cell glutamine metabolism, we found that circ_0000808 might promote glutamine metabolism process by regulating miR-1827/SLC1A5 network. Therefore, we confirmed that circ_0000808/miR-1827/SLC1A5 axis regulated NSCLC progression through mediating glutamine metabolism.

In conclusion, our study suggested a new regulatory axis for regulating NSCLC progression. Our results revealed that circ_0000808, as a miR-1827 sponge, accelerated the proliferation, migration, and invasion of NSCLC by promoting glutamate metabolism through upregulating SLC1A5. Our study revealed the potential molecular mechanism by which circ_0000808 regulated NSCLC development for the first time. Our study is the first to reveal the potential of circ_0000808 as a therapeutic target for NSCLC, providing a new idea for molecular targeted therapy of NSCLC. Of course, there are some limitations to our study. Due to the limited clinical sample size, we cannot be certain that all subtypes of NSCLC have the same outcome. More sample sizes need to be collected in the future to further confirm our conclusions.

## Supplementary Information

**Additional file 1: Supplementary Fig. 1.** The expression of ZEB1 and Vimentin. (A) WB analysis was used to examine the expression of ZEB1 and Vimentin in NSCLC cells transfected with sh-NC or sh-circ_0000808. (B) The expression of ZEB1 and Vimentin in tumor tissues of each group was measured by WB analysis. ***P < 0.001.

**Additional file 2: Supplementary Fig. 2.** The representative images of Fig. 4D (A), 4E (B), 4F (C), 4G (D) and 4H (E).

**Additional file 3: Supplementary Fig. 3.** The representative images of Fig. 6E (A), 6F (B), 6G (C), 6H (D) and 6I (E).

## Data Availability

Not applicable
