# Supplementary figures and images for: Circ_0000808 promotes the development of non-small cell lung cancer by regulating glutamine metabolism via the miR-1827/SLC1A5 axis
Source: World J Surg Oncol. 2022 Oct 3;20:329. doi: 10.1186/s12957-022-02777-x (PMC9528172; doi:10.1186/s12957-022-02777-x)

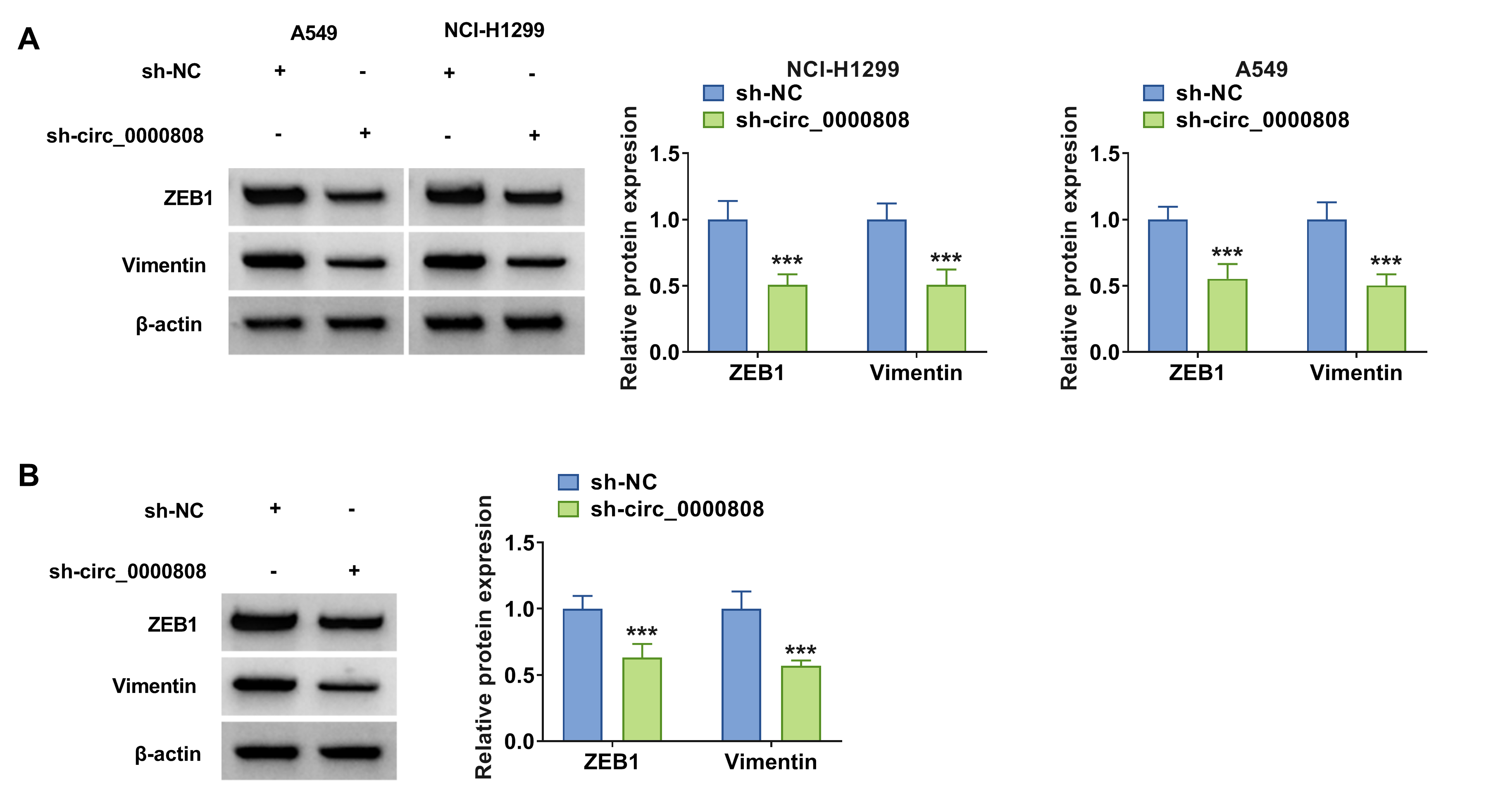

Supplement: Supplementary file 1 — Additional file 1: Supplementary Fig. 1. The expression of ZEB1 and Vimentin. (A) WB analysis was used to examine the expression of ZEB1 and Vimentin in NSCLC cells transfected with sh-NC or sh-circ_0000808. (B) The expression of ZEB1 and Vimentin in tumor tissues of each group was measured by WB analysis. ***P < 0.001. [file 12957_2022_2777_MOESM1_ESM.tif]

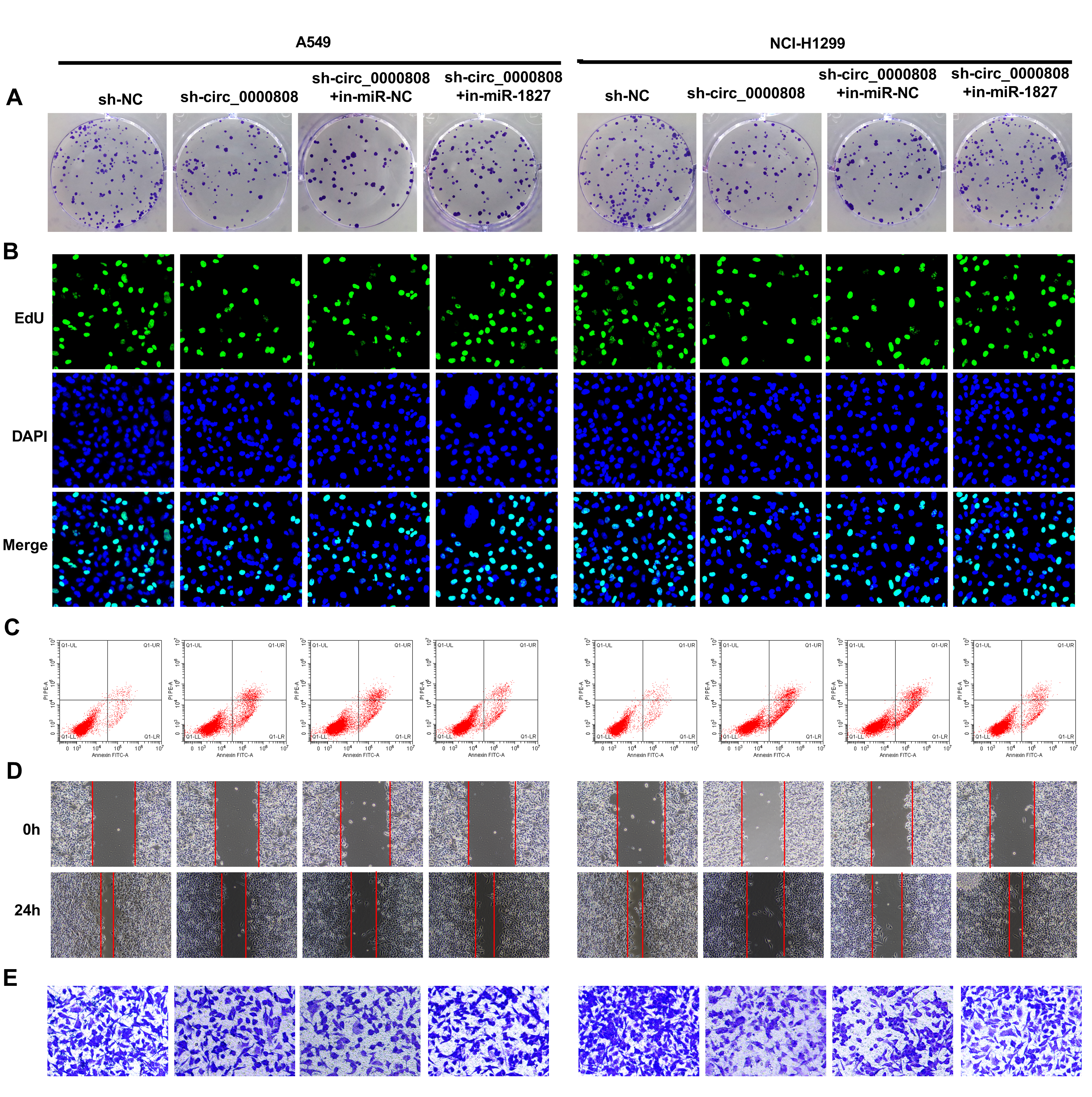

Supplement: Supplementary file 2 — Additional file 2: Supplementary Fig. 2. The representative images of Fig. 4D (A), 4E (B), 4F (C), 4G (D) and 4H (E). [file 12957_2022_2777_MOESM2_ESM.tif]

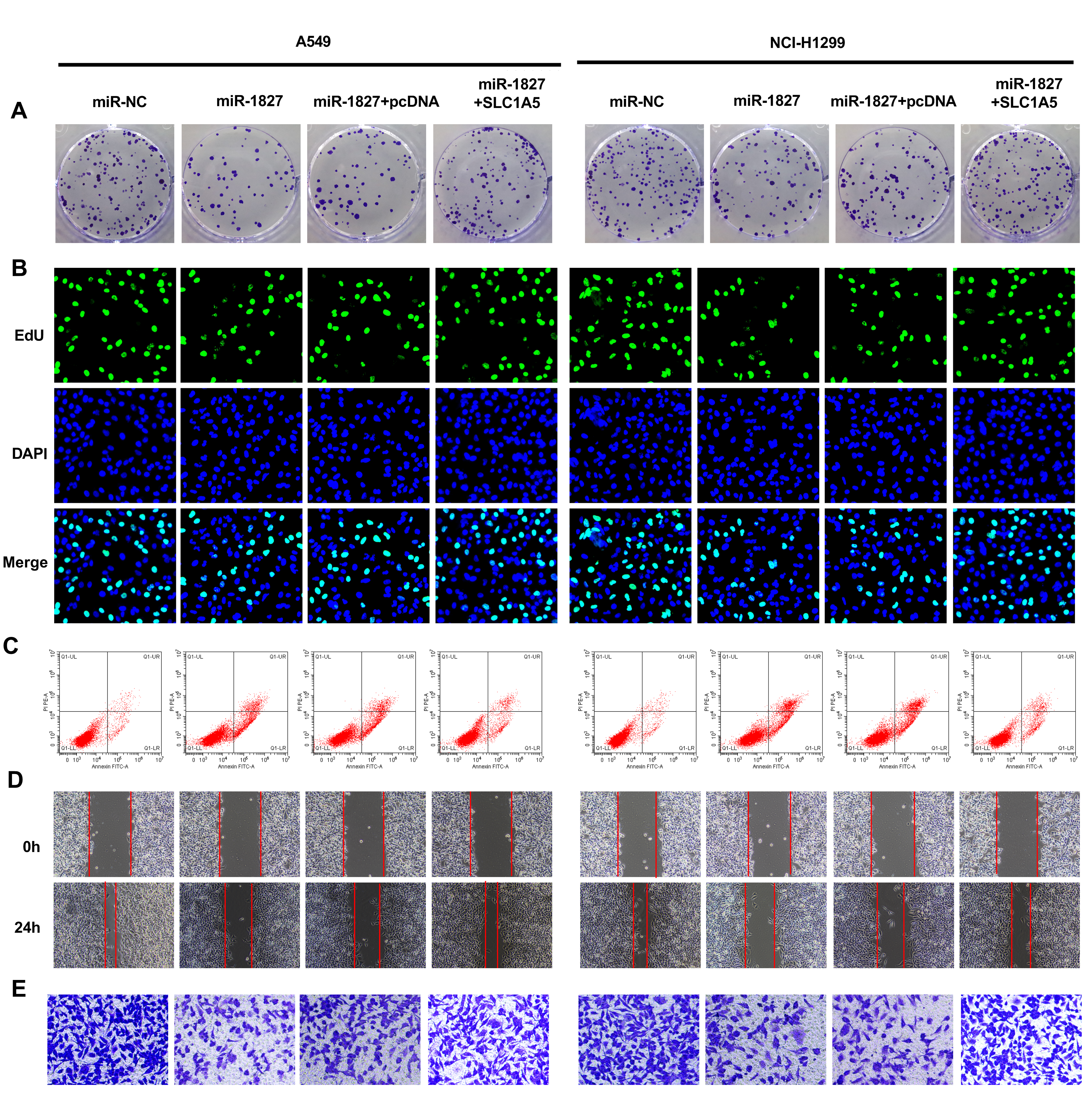

Supplement: Supplementary file 3 — Additional file 3: Supplementary Fig. 3. The representative images of Fig. 6E (A), 6F (B), 6G (C), 6H (D) and 6I (E). [file 12957_2022_2777_MOESM3_ESM.tif]
